# CX-4945: the protein kinase CK2 inhibitor and anti-cancer drug shows anti-fungal activity

**DOI:** 10.1007/s11010-017-3068-z

**Published:** 2017-05-13

**Authors:** Maciej Masłyk, Monika Janeczko, Aleksandra Martyna, Konrad Kubiński

**Affiliations:** Department of Molecular Biology, Institute of Biotechnology, The John Paul II Catholic University of Lublin, ul. Konstantynów 1i, 20-708 Lublin, Poland

**Keywords:** CX-4945, Protein kinase CK2, Yeast, Anti-fungal agents, Antitumor agents

## Abstract

CX-4945 is a selective inhibitor of protein kinase CK2 exhibiting clinical significance. Its antitumor properties arise from the abrogation of CK2-mediated pro-survival cellular pathways. The presented data reveal the influence of CX-4945 on the growth of yeast cells showing variable potency against *Saccharomyces cerevisiae* deletion strains with different contents of CK2 subunits. The catalytic subunit CK2α appears to sensitize yeast to the CX-4945 action. Moreover, the compound suppresses hyphal growth and cell adhesion of *Candida albicans*, thereby abolishing some hallmarks of invasiveness of the pathogen. It is known that cancer patients are more prone to fungal infections. Our data unveil the dual-activity of CX-4945; when used in anti-cancer therapy, it may simultaneously prevent cancer-associated candidiasis.

## Introduction

CX-4945 5-(3-chlorophenylamino)benzo[c] [2, 6] naphthyridine-8-carboxylic acid, also known as Silmitasertib, is an ATP- competitive inhibitor of protein kinase CK2. It is a highly selective, orally administered small molecule studied in different types of human cancer research [1]. It has been shown that the mechanism of antitumor activity is mediated through inhibition of CK2-dependent processes required for maintenance of the phenotype of cancer cells [2]. It has been evidenced that CX-4945 attenuates the PI3K/Akt signaling pathway by suppressing the phosphorylation of Akt and other crucial downstream mediators of the pathway such as p21 [3]. Furthermore, it selectively induces apoptosis in cancer cells and exhibits antiproliferative and anti-angiogenic activity against abnormal cells. CX-4945 exerts its antitumor effects in a wide range of cancer cell types, in which CK2 is overexpressed, such as lung, breast, and prostate cancer cells [2, 3]. Moreover, CX-4945 is the first inhibitor of CK2 that has been qualified for human clinical trials, has successfully completed phase I, and is currently in phase II for cholangiocarcinoma treatment, which granted it an Orphan Drug status for cholangiocarcinoma in the USA in January 2017. Human clinical characterization of CX-4945 as a single agent in solid tumors and multiple myeloma has shown its promising pharmacokinetic, pharmacodynamics, and safety profiles [4]. In addition, besides its antitumor activity, CX-4945 may be developed as a new therapeutic agent for pathologies correlated with CK2 dysregulation, such as inflammatory disorders, neurodegenerative diseases, and viral or parasitic infections [5, 6]. Considering all above-mentioned characteristics, CX-4945 is assumed to have a significant role in novel therapeutic strategies for treatment of various types of cancer in the future.

CK2 (casein kinase II) is a highly pleiotropic and constitutively active serine/threonine protein kinase regulating a broad spectrum of major cellular processes by reversible phosphorylation of proteins [7, 8]. It appears in a heterotetrameric form, composed of two catalytic subunits (α and α’) and two regulatory β-subunits, or in a monomeric form. Depending on the organism, different isoenzymatic forms of the catalytic subunit of CK2 have been identified [9, 10]. Human CK2 has two well-characterized isoforms of the catalytic subunit, designated as CK2α and CK2α’, and a third one, i.e., CK2α”, which is highly expressed in the liver [11]. Protein kinase CK2 participates in the regulation of a wide array of physiological and pathological processes in the cell, such as cancer development, transcriptional regulation, signal transduction, proliferation, cell cycle control, and apoptosis [12].

Here we present for the first time the influence of CX-4945 on single-celled lower eukaryotes—yeast *Saccharomyces cerevisiae* and *Candida albicans*. To date, there are many reports describing the role of CK2 in yeast, and the influence of CK2 has been shown to govern virulence in *C. albicans* [9, 13–15]. Moreover, an increasing number of reports support the view that there is an association between cancer and fungal infections [16].

## Materials and methods

### Microbial strains

CX-4945 was screened for its in vitro anti-fungal activity against the standard strains: *C. albicans* ATCC 10231, *S. cerevisiae*: wild-type strain BMA64-1A, and a deletion mutant lacking CK2α (YIL035c), and CK2α′ (YOR061w). Additionally, CK2α (YIL035c) was transformed with the shuttle vector pYES2C/T::CKA1.

*Microbial susceptibility* The yeast strains were inoculated in Sabouraud Dextrose liquid medium (Biocorp, Poland) and incubated at 30 °C with vigorous shaking (200 rpm) for 24 h before performing the Minimal Inhibitory Concentration (MIC) and Minimal Fungicidal Concentration (MFC) tests. MIC was determined with the microbroth dilution method. Microbial cell suspensions at initial inoculums of 3 × 103 colony forming units per ml in Sabouraud Dextrose Broth were exposed to the tested compound at adequate concentrations (range 0.001–2 mg/ml) for 48 h at 30 °C. MIC was the lowest concentration of the compound that inhibited the visible growth of the microorganism. After MIC readings, 10 µl aliquots were removed from the wells corresponding to MIC, 2 × MIC, and 4 × MIC and spread on SDA (Sabouraud Dextrose Agar) petri dishes. The plates were incubated at 30 °C and the fungal colonies grown were counted after approximately 2 days of incubation. The MFC was defined as the lowest drug concentration from which ≤1 colony was visible on the agar plate. The experiments were performed in triplicate.

### Hyphal growth of *Candida*

The effect of CX-4945 on the hyphal growth of the *Candida* reference strain was evaluated using Spider medium. *Candida* cells were grown overnight in Sabouraud broth medium (Biocorp, Poland) in a shaker at 180 rpm and 37 °C. At the late exponential growth phase, the yeasts cells were harvested using a microcentrifuge (Polygen 1-15PK, Poland) at 2300 g for 15 min. The yeast cells were washed twice with phosphate buffered saline, pH 7.2, and resuspended in PBS to reach an optical density (OD_600_) of 0.38 (107 cells/ml). 100 µl of the suspension containing 107 cells/ml was used for the assays. *Candida* cells were grown on Spider medium plates containing 10% fetal bovine serum (FBS), supplemented with or without CX-4945 at the concentration of MIC/10. The plates were incubated at 37 °C for 36 h. The morphology of *Candida* colonies was inspected under a light microscope and imaged using a digital camera.

### In vitro biofilm formation assay

Biofilm assays were performed using a microtiter plate-based method. Sabouraud Dextrose Broth medium (Biocorp, Poland) was used to prepare the bacterial inoculum. To evaluate the effects of the CX-4945 on the initial biofilm formation, the *C. albicans* strain was grown in Saburoud medium in a shaker (250 rev/min) at 37 °C for 24 h. Then, the yeast culture was diluted (1:100) in the same medium containing subinhibitory concentrations (1/2 MIC, 1/4 MIC, 1/8 MIC, and 1/16 MIC) of the compound. A volume of 200 µl of this mixture was inoculated into each well of a 96-well inert polystyrene microtiter plate. After incubation of the microplate at 37 °C for 24 h, the supernatants were removed, and the biofilm was washed once with distilled water. It was dried and fixed at 65 °C for 1 h. Finally, the wells were stained with crystal violet and washed, and the absorbance at 570 nm was determined using a microplate reader (BIOTEK SYNERGY HT). For examining the possible effects of the compound solvent in biofilm formation, 1% DMSO was used in the place of the compounds tested in the experiment.

To analyze the effect of the active compound on the mature biofilm, the growth of *C. albicans* biofilm was induced as described above, but in the absence of the compounds, and was incubated for 24 h. Then, the supernatants were gently removed, and the concentrations that were 1/2 MIC, 1/4 MIC, 1/8 MIC, and 1/16 MIC for the compound prepared in Sabouraud broth were added to each well of the microtiter plate. After 24 h of incubation, the assay was read as described above. All experiments were performed at least three times with four replicates each. 1% farnesol was used as a positive control.

## Results and discussion

**Fig. 1:**
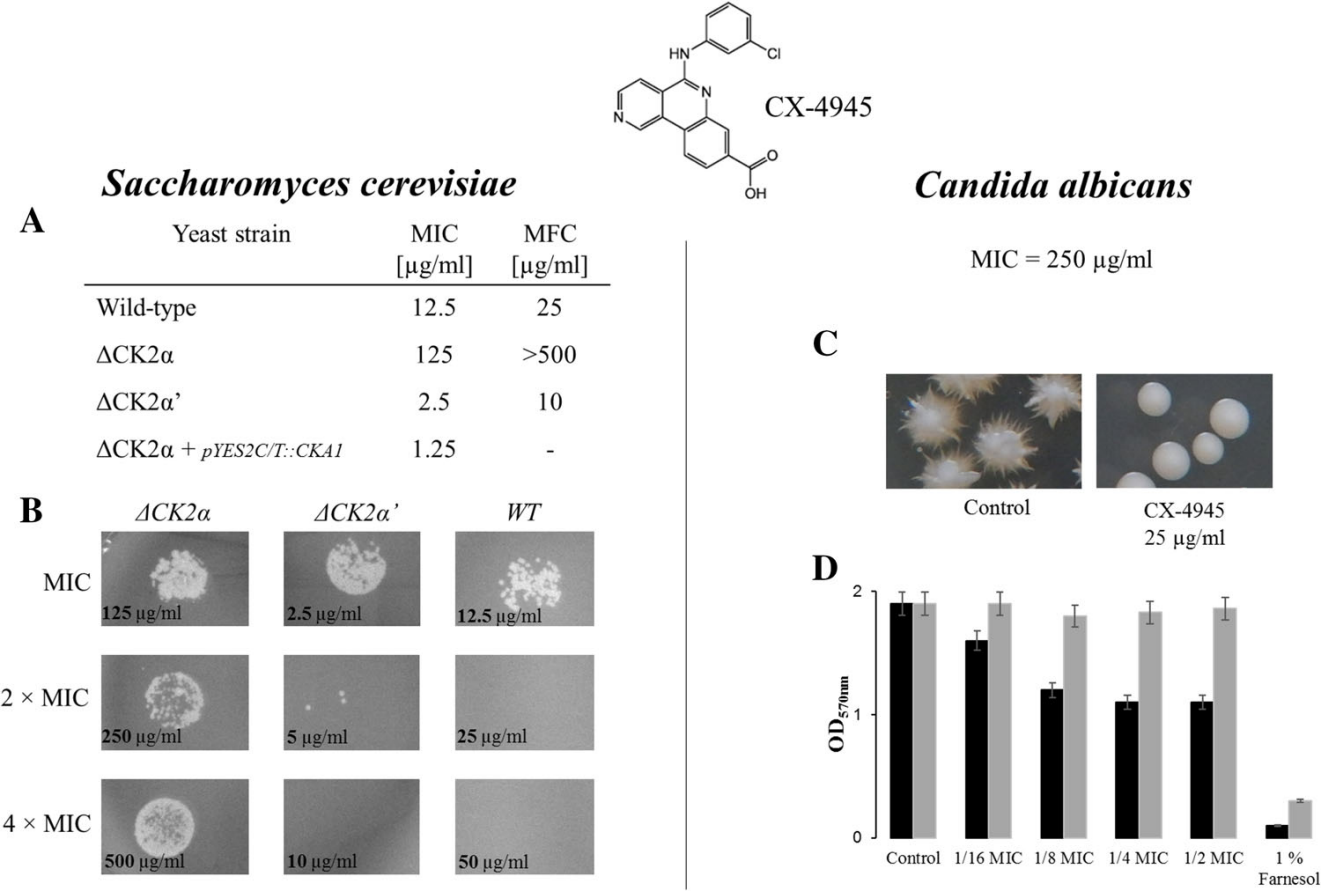
**a** MIC values for CX-4945 against *S. cerevisiae* strains, **b** Macroscopic view of the growth of different *S. cerevisiae* strains. After the MIC assay, the cells from appropriate wells corresponding to MIC, 2 × MIC, and 4 × MIC, were cultured on SDA medium. **c** Macroscopic view of *C. albicans* colonies grown on agar medium supplemented with FSB (hyphal inductor) and DMSO (control) or CX-4945. **d** Effect of CX-4945 on biofilm formation of Candida cells. The *black bars* indicate the influence of the compound on the adhesion phase during biofilm formation and the *gray bars* reflect the influence of CX-4945 on established, mature biofilm. Farnesol was used as a positive control

Since CK2 is essential for yeast viability, we verified the possible effect of CX-4945 on *S. cerevisiae* and *C. albicans*. The MIC (minimal inhibitory concentration) values of 12.5 and 250 µg/ml determined for *S. cerevisiae* and *C. albicans*, respectively, revealed a striking discrepancy between our results and the previously reported EC_50_ value for human cells [1]. When tested on 43 cancer cell lines, the mean EC_50_ across all cell lines was 2 µg/ml (5.5 µM). The only explanation that arises is the difficulty of CX-4945 in penetration of the yeast cell wall. We previously reported that different yeast CK2 isoforms αα’ββ’, α’α’ββ’, ααββ’, and free catalytic α’ may differ in susceptibility to inhibitors [17, 18]. We employed two *S. cerevisiae* strains lacking the CK2α or CK2α’ subunit. The MIC values of 125 and 2.5 µg/ml for the Δα and Δα’ strains, respectively, showed an intriguing difference in their sensitivity towards CX-4945. This clearly shows that the susceptibility of yeast cells to the Silmitasertib action depends on the content of the CK2 subunits and the holoenzyme composition. The transformation of the Δα strain with the shuttle vector carrying the gene encoding the CK2α subunit, restoring the native CK2 content, sensitized the yeast to CX-4945 (MIC = 1.25 µg/ml) (Fig. 1a). Previous in vitro studies on human CK2 isoforms revealed that CX-4945 does not discriminate between different CK2 forms [1, 3]. The compound inhibits the human CK2 holoenzyme as well as free catalytic subunits with the same potency (K_i_ = 0.38 nM). To date, these results have not been verified in in vivo studies.

Besides MIC values, which reflect the influence of the compound on *S. cerevisiae* cell growth, the fungicidal activity of CX-4945 was assayed. The MFC (minimal fungicidal concentration) values of 25, > 500, and 10 µg/ml determined for the wild-type, Δα, and Δα’ strains, respectively, indicate that CK2α sensitizes the yeast cells to the CX-4945 action (Fig. 1b). The most sensitive strain Δα’ expresses solely the CK2α subunit, and thus the cellular CK2 activity is provided only by the holoenzyme ααββ’.

In order to verify whether CX-4945 can abrogate cellular functions of CK2 in yeast, we employed human pathogenic fungus *C. albicans*. Engagement of CK2 in *Candida* virulence was previously reported [13, 14]. *Candida albicans* appeared to be more resistant to Silmitasertib in comparison with *S. cerevisiae*, with a MIC value of 250 µg/ml. Very recently, we have reported that a natural CK2 inhibitor emodin abrogates hyphal growth of *C. albicans* [19]. In this study, we observed a similar effect in the case of the clinical CK2 inhibitor, where the CX-4945 concentration of 1/10 MIC inhibited yeast-to-hypha transition (Fig. 1c). The phenomenon of hyphal formation is directly involved in *Candida*-mediated infection [20]. Lo and coworkers showed that mutants that are unable to form hyphae are attenuated in virulence [21]. Another fungal invasion factor is the ability of microorganisms to form biofilm, which in its mature form protects the pathogen from antimicrobial agents and host immune factors, and contributes to poor prognosis [22, 23]. Although CX-4945 did not influence mature biofilm, it effectively inhibited the adhesion phase of the biofilm formation process by 42% at the concentration of 62.5 µg/ml (1/4 MIC) (Fig. 1d).

In summary, the CK2 inhibitor CX-4945 has been recently designated by the U.S. Food and Drug Administration (FDA) as an Orphan Drug for the treatment of cholangiocarcinoma. Our results have shown that the compound inhibits the growth of *S. cerevisiae* discriminating between yeast cell variants carrying different isoforms of protein kinase CK2. Moreover, CX-4945 can suppress the growth of an opportunistic fungal pathogen *C. albicans*. It effectively prevents the fungus from hyphal growth as well as adhesion. Since, the opportunist pathogen takes advantage of the immunosuppressed state of patients, particularly caused by chemotherapy, the application of CX-4945 in targeted chemotherapy may prevent possible cancer-associated candidiasis.
